# Correlation of zinc finger protein 2, a prognostic biomarker, with immune infiltrates in liver cancer

**DOI:** 10.1042/BSR20203115

**Published:** 2021-01-22

**Authors:** Lei Sun, Yaru Lin, Guichun Wang, Lin Zhang, Liangchang Hu, Zhong Lu

**Affiliations:** 1 Clinical College of Weifang Medical University, Weifang, 261053, China; 2 Department of oncology, Affiliated Hospital of Weifang Medical University, Weifang, 261031, China

**Keywords:** bioinformatics mining, immune infiltration, liver cancer, TCGA database, ZIC2

## Abstract

Purpose: The expression and clinical value of zinc finger protein 2 gene (*ZIC2*) in hepatocellular carcinoma (HCC) were analyzed by mining gene information from The Cancer Genome Atlas (TCGA) and Gene Expression Omnibus (GEO) databases.

Methods: Gene chip data sets were retrieved from GEO and TCGA and screened for differentially expressed genes in HCC. Gene expression profile interaction analysis (GEPIA) and Kaplan–Meier curves were used to analyze the relationship between differentially expressed genes (DEGs) and survival and prognosis in patients with HCC. Moreover, the Genecards database was used to extract *ZIC2*-related proteins and to analyze the physiological process of protein enrichment. Furthermore, the relationships between *ZIC2* gene and tumor cell immune invasion and that between immune cell infiltration and the 5-year survival rate were studied using the tumor immune evaluation resource (TIMER) database.

Results: Datasets from GEO and TCGA revealed that *ZIC2* was differentially expressed in HCC tissues and normal tissues (*P*<0.05). High *ZIC2* expression was associated with overall survival (OS) and progress-free survival in HCC patients. Overall, 25 *ZIC2* related proteins, including Gli3, PRKDC, and rnf180 were identified and protein enrichment analysis indicated these were associated with four types of cell components, six types of cell functions, and eight types of biological processes. *ZIC2* was positively correlated with immune infiltration cells in patients with HCC, and higher expression of *ZIC2* mRNA CD4+T cells is associated with a better 5-year survival.

Conclusion: *ZIC2* gene may be used as an immune response marker in liver cancer to predict the prognosis of HCC.

## Introduction

Zinc finger protein ZIC2 is encoded by the *ZIC2* gene and can interact with DNA and proteins [1]. The expression of *ZIC2* may promote cell proliferation and inhibit cell apoptosis [2]. *ZIC2* is a member of the zinc finger of cerebellum (ZIC) family of C2H2 zinc finger proteins. The encoded protein acts as a transcription inhibitor and can regulate the tissue-specific expression of dopamine receptor D1. Gene ontology (GO) annotations related to *ZIC2* gene include DNA-binding transcription factor activity and chromatin DNA binding [3]. Recent studies have reported that *ZIC2* functions as an oncogene in various cancers [4]. Numerous studies have found that the *ZIC2* gene is expressed abnormally in a various solid tumors including breast cancer [5], nasopharyngeal cancer [6], and cervical cancer [7]. Tumor immunotherapy, characterized by immunosuppressive checkpoint inhibitors, has represented an important breakthrough, and has thereby introduced novel therapies for the treatment of cancer in recent years [8]. The emergence of immunotherapy provides a novel therapeutic approach, which has been approved for the treatment of liver cancer, and has achieved significant results, whereas the objective response rate of Programmed Death 1/ Programmed Cell Death-Ligand 1(PD-1/PD-L1) antibody treatment alone rarely exceeded 40%, while that of nivolumab and pembrolizumab in HCC was less than 20% [9]. Therefore, comprehensive analysis of gene mutations, gene expression, cytokines, chemokines, and tumor infiltrating cells is urgently required in the era of precision medicine. Further, the lack of immunotherapy research dedicated to patients with liver cancer requires formulation of a precise personalized immunotherapy scheme, effectively evaluation and prediction of the potential curative effect of immunotherapy, and thus adoption of a combined treatment strategy. Although studies on the expression of *ZIC2* in hepatocellular carcinoma (HCC) are available, only a few studies have reported the correlation between *ZIC2* gene and tumor infiltrating immune cells in the tumor microenvironment.

Liver cancer is one of the 10 major malignant tumors and the fourth leading cause of cancer-related death worldwide. According to the Global Malignant Tumor Status Report [10], the global incidence of liver cancer was 841,000, ranking sixth highest in the incidence of malignant tumors, of which the number of new cases in China accounted for 46.6% of the global incidence of liver cancer. Liver cancer deaths worldwide amount to approximately 781,000, ranking fourth in the malignant tumor death spectrum, with 47.1% occurring in China [11]. In most cases, the disease is diagnosed in the middle or late stages; hence, its prognosis becomes difficult as the tumor cannot be removed, and the overall survival (OS) rate of these patients is only a few months [12]. The development of various targeted drugs has prolonged the survival of patients, and, in particular, sorafenib has become the main first-line treatment option for advanced HCC, but patients who use sorafenib for first-line treatment do not survive for more than 1 year [13,14]. Molecular mechanisms of liver cancer progression include epithelial–mesenchymal transformation, tumor–stromal interaction, tumor microenvironment, cancer stem cells, and senescence bypass, and in addition may also involve circulating tumor cells and immunomodulatory and neuroregulatory mechanisms. These complex mechanisms may involve processes such as cell cycle regulation and signal transduction, and thus reflect the functional interaction of various genes in multiple steps [15,16].

Screening gene networks for changes related to tumor formation and progression may lead to the identification of new biomarkers for future diagnosis and treatment of liver cancer. Therefore, it is of great clinical significance to study the expression of *ZIC2* gene in liver cancer, the clinical prognosis of patients with liver cancer, and the potential relationship and mechanisms involving *ZIC2* gene and tumor immune system interactions. Meanwhile, this research can also help clinical oncologists to understand the underlying mechanisms of tumorigenesis and progression from a molecular biology perspective.

We have identified alternative expression of ZIC family members in various cancer types [17]. Some studies have reported that *ZIC2* functions as an oncogene in cancers, whereas others have revealed that the decreased expression of *ZIC2* promotes tumorigenesis [18]. Therefore, the level of expression of different ZIC family members may depend on the type of cancer [19]. For example, *ZIC2* is highly expressed in HCC [20], bone cancer, and cervical cancer, whereas it is less expressed in breast cancer [21], thyroid cancer [22], and gastric cancer [23]. Recently, several studies have focused on the role of ZIC family proteins in tumorigenesis.

We comprehensively analyzed the differential expression of the *ZIC2* gene and its relationship with the prognosis of cancer patients using bioinformatics databases in the present study. These databases include The Cancer Genome Atlas (TCGA), Gene Expression Omnibus (GEO), Gene expression profile interaction analysis (GEPIA), and Kaplan–Meier plots. Moreover, we used the TIMER database to study the correlation between *ZIC2* and tumor infiltrating immune cells in the liver cancer microenvironment. The findings of this report support the involvement of *ZIC2* in immune interaction mechanisms underlying liver cancer tumors.

## Materials and methods

### Bioinformatics analysis and datasets

Subject and gene information were obtained from GEO (http://www.ncbi.nlm.nih.gov/geo/)and TCGA (https://www.cancer.gov/about-nci/organization/ccg/research/structural-genomics/tcga) databases. Gene expression profiles were searched and selected from the GEO and TCGA databases. Differentially expressed genes (DEGs) between HCC and noncancerous samples were screened by GEO2R.

### GEPIA database search

GEPIA (http://gepia2.cancer-pku.cn) is a newly developed interactive web server for analyzing the RNA sequencing expression data of 9736 tumors and 8587 normal samples from the TCGA and the GTEx projects, using a standard processing pipeline. GEPIA provides customizable functions such as tumor/normal differential expression analysis, profiling according to cancer types or pathological stages, patient survival analysis, similar gene detection, correlation analysis, and dimensionality reduction analysis. This tool was developed by Zefang Tang, Chenwei Li, and Boxi Kang of Zhang Lab (http://cancer-pku.cn/), Peking University, Beijing, China. The expression of the *ZIC2* gene was analyzed online through the GEPIA database comparing liver cancer and normal tissues. The screening conditions were set as follows: select Expression on Box Plots, Gene: ZIC2, Log2 fold change (FC) ≥ 1, *P*<0.01, Cancer type: liver HCC (LIHC). The statistical significance is annotated by the number of stars (*: *P*-value<0.05; **: *P*-value <0.01; ***: *P*-value <0.001).

### TCGA data

Whole-genome RNA-seq datasets relative to HCC in TCGA were downloaded from the website: https://www.cancer.gov/types/liver. Samples in three groups were stratified according to their *ZIC2* gene levels (low, intermediate, and high). The 25th and 75th percentiles were used as cut-off values. The Benjamini–Hochberg procedure was used to calculate the false discovery rate (FDR) and up-regulated genes were chosen for GO analysis using http://metascape.org/gp/index.html#/main/step1.

### Kaplan–Meier plotter database analysis

The relationship between OS, progress-free survival (PFS), and *ZIC2* gene expression was analyzed online using the liver cancer data set of Kaplan–Meier Plotter database (http://kmplot.com/analysis/). The screening conditions were set as: (1) start KM Plotter for liver cancer; (2) gene symbol: ZIC2; (3) survival: OS, PFS.

### Genecards database analysis

The Genecards database (https://www.genecards.org/) was used to find information and related proteins of the *ZIC2* gene and to construct a network map of these proteins.

### TIMER database analysis

The TIMER web server (https://cistrome.shinyapps.io/timer/) is a comprehensive resource for systematical analysis of immune infiltrates across diverse cancer types. The abundance of six immune infiltrates (B cells, CD4+ T cells, CD8+ T cells, neutrophils, macrophages, and dendritic cells) is estimated by the TIMER algorithm. The TIMER web server allows users to input function-specific parameters, with resulting figures dynamically displayed to conveniently access the immunological, clinical, and genomic features of the tumor. The TIMER database can systematically analyze the immune infiltration of different cancer types, setting the screening conditions as: (1) gene symbol: *ZIC2*; (2) cancer types: (LIHC); immune infiltrates: B Cell, CD8+T Cell, CD4+T cell, macrophage, neutrophil, and dendritic cell.

### Statistical analysis

SPSS version 22.0 software was used for statistical analysis. The Student‘s *t*-test was used for comparisons between liver cancer and normal tissues. Kaplan-Meier analysis and the Log-rank test was used for survival analysis.

## Results

### Screening of DEGs

**Figure 1:**
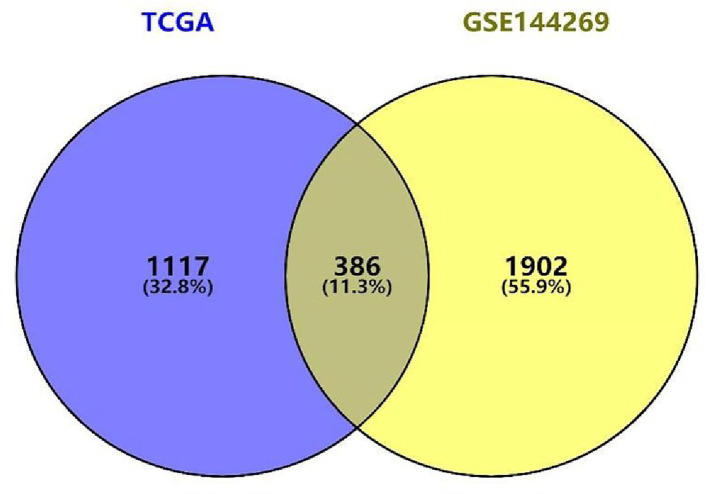
The differentially expressed genes (DEGs) were identified in the mRNA expression profile data set. Venn diagram of DEGs (*P*<0.001 and | LogFC | > 2.5)

In total, 1503 and 2288 DEGs were identified from GSE144269 and TCGA datasets, and 386 genes were found to be common to both data sets. Interestingly, the trend of *ZIC2* gene expression was similar for both datasets (Figure 1).

### ZIC2 mRNA expression level in liver cancer

**Figure 2:**
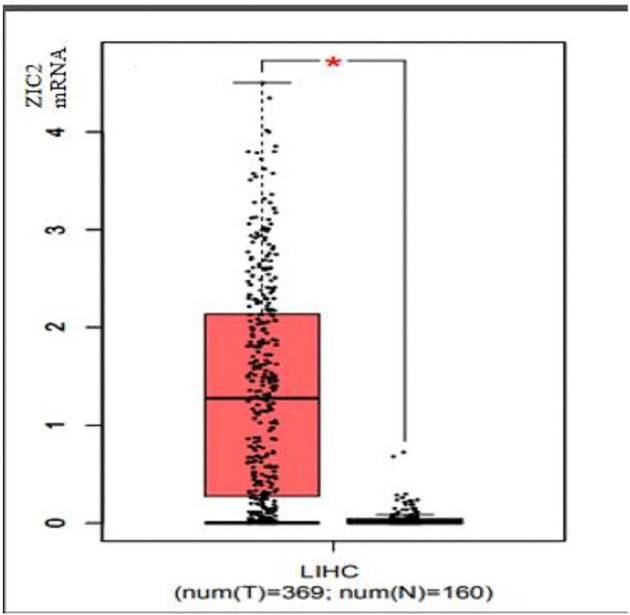
Different expression of *ZIC2* mRNA in hepatocellular carcinoma compared with normal liver tissue (*: *P*-value<0.05).

The analysis results of the GEPIA database revealed that compared with normal tissues, the level of mRNA expression of *ZIC2* gene in liver cancer tissues was significantly higher (*P*<0.05, Figure 2).

### ZIC2 up-regulation promotes liver cancer growth via changes in the cell cycle

**Figure 3:**
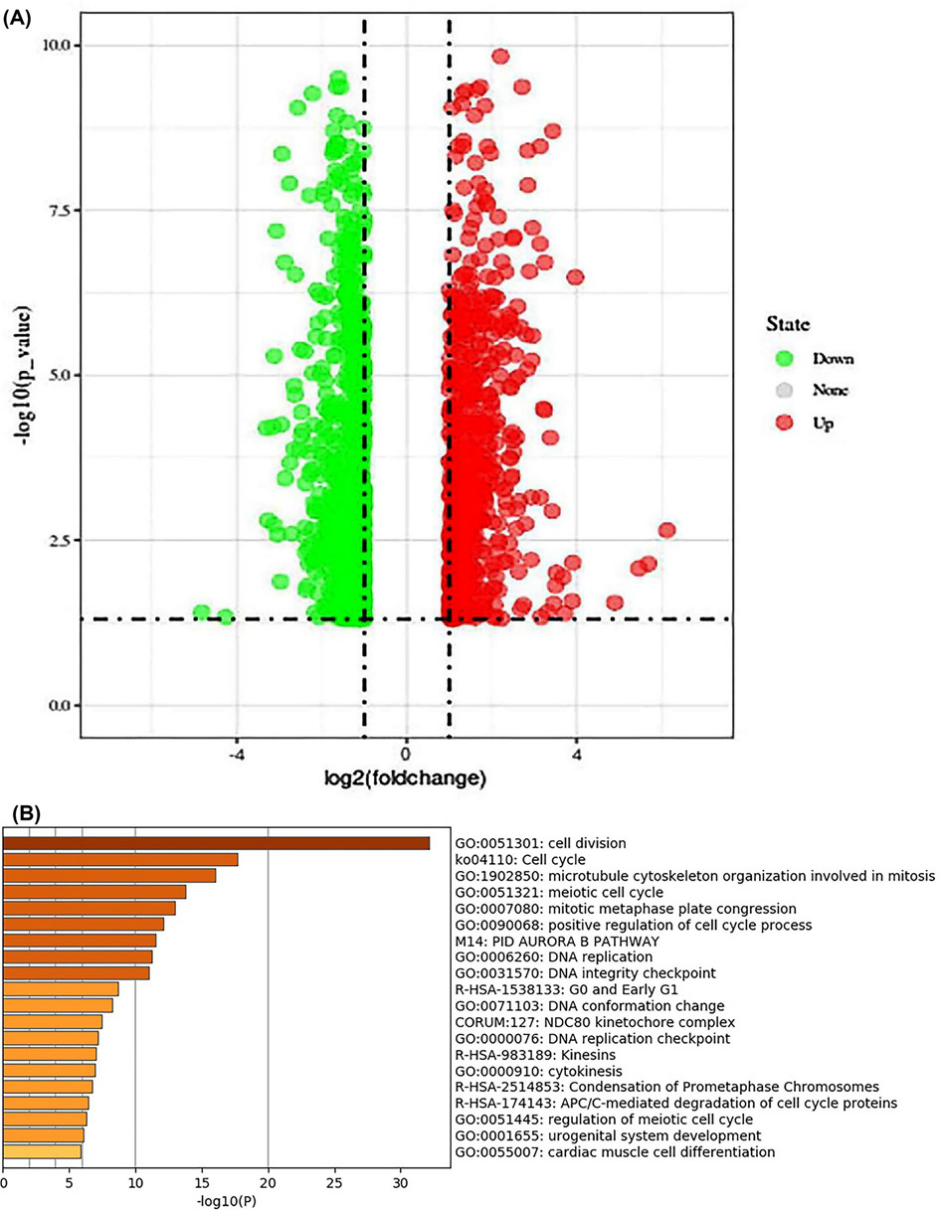
Enrichment analysis of *ZIC2* overexpression promoting hepatoma cell growth (**A**) Differentially expressed genes (DEGs) in 25% high versus 25% low *ZIC2* mRNA expression levels in liver cancer. (**B**) Gene oncology (GO) analysis results were listed. As illustrated, DEGs were primarily enriched in cell division and cell cycle functions.

To further reveal the function of *ZIC2* in HCC, we assessed the RNA-seq data of HCC patients in TCGA and GEO datasets having different levels of *ZIC2* expression. In total, 386 highly expressed genes were found in patients having higher *ZIC2* versus (Figure 3A). GO enrichment analysis indicated that high *ZIC2* expression in patients with liver cancer might be associated mainly with the cell cycle (Figure 3B). These findings indicated that ZIC2 up-regulation could promote liver cancer growth presumably via cell cycle alteration and regulation of cell division.

### Survival analysis results based on the Kaplan–Meier plotter database

**Figure 4:**
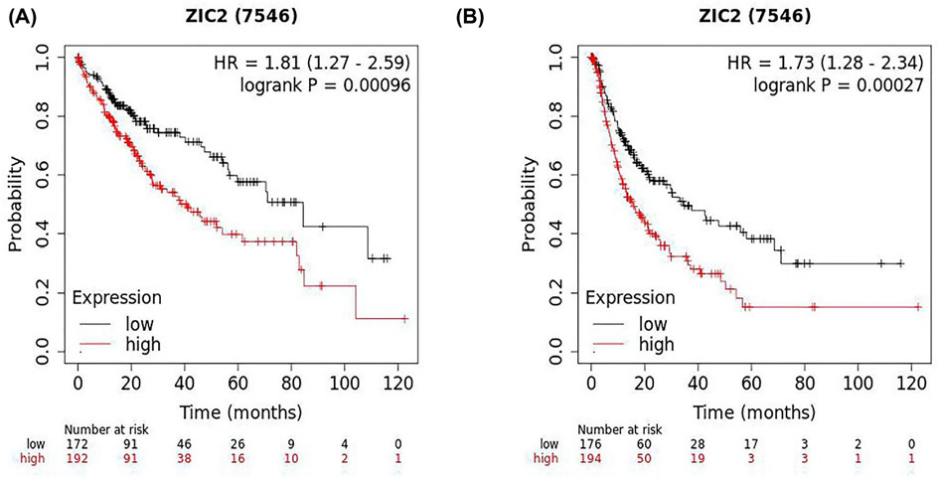
The poor prognosis of liver cancer is related to the expression of *ZIC2* (**A**) OS HR = 1.81, 95% CI = 1.27–2.59, Logrank *P* = 0.00096. (**B**) PFS HR = 1.73, 95% CI = 1.28–2.34, Logrank *P* = 0.00027.

To further study the prognostic potential of *ZIC2* in liver cancer, we used the Kaplan–Meier plotter tool to evaluate the prognostic value of *ZIC2*. Poor prognosis of liver cancer was associated with higher *ZIC2* expression (OS HR = 1.81, 95% confidence interval [CI]= 1.27–2.59, Logrank *P*=0.00096, Figure 4A; PFS HR = 1.73, 95% CI = 1.28–2.34, Logrank *P*=0.00027, Figure 4B), and the differences are statistically significant.

### ZIC2 gene related protein network map

**Figure 5:**
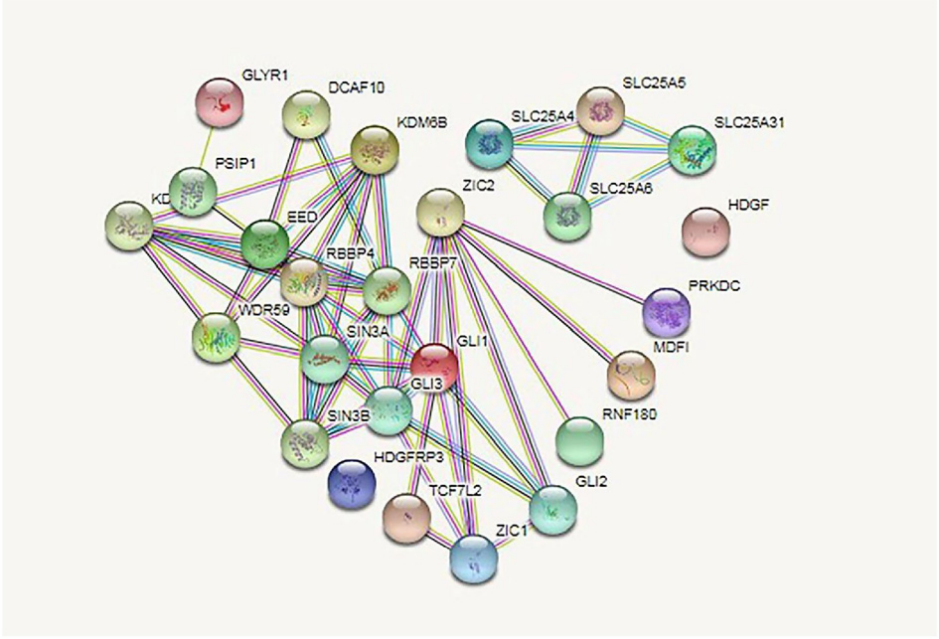
The network maps of *ZIC2* related proteins and 25 major related proteins of the *ZIC2* gene

Genecards were used to obtain the network maps of *ZIC2* related proteins and to analyze the relationship of 25 major proteins associated with the *ZIC2* gene, such as GLI3, PRKDC, and RNF180. The results of protein enrichment analysis are mainly reflected in four types of cellular components, six types of molecular functions, and eight types of biological processes (Figure 5). This analysis mainly revealed the involvement of *ZIC2* gene expression in physiological processes such as regulation of transcription, cell differentiation, and positive regulation of DNA-binding transcription factor activity (Table 1).

**Table 1:** Go function annotation of *ZIC2* and related genes

Categories	Go ID	Function
Molecular function	GO:0000977	RNA polymerase II regulatory region sequence-specific DNA binding
	GO:0000981	DNA-binding transcription factor activity, RNA polymerase II-specific
	GO:0003677	DNA binding
	GO:0003700	DNA-binding transcription factor activity
	GO:0031490	Chromatin DNA binding
	GO:0046872	Metal ion binding
Biological process	GO:0006357	Regulation of transcription by RNA polymerase II
	GO:0007417	General nervous system development
	GO:0007420	Brain development
	GO:0007601	Visual perception
	GO:0030154	Cell differentiation
	GO:0045892	Negative regulation of transcription DNA-templated
	GO:0045893	Positive regulation of transcription DNA-templated
	GO:0051091	Positive regulation of DNA-binding transcription factor activity

### The expression of ZIC2 in liver cancer was correlated with the level of immune infiltration

**Figure 6:**
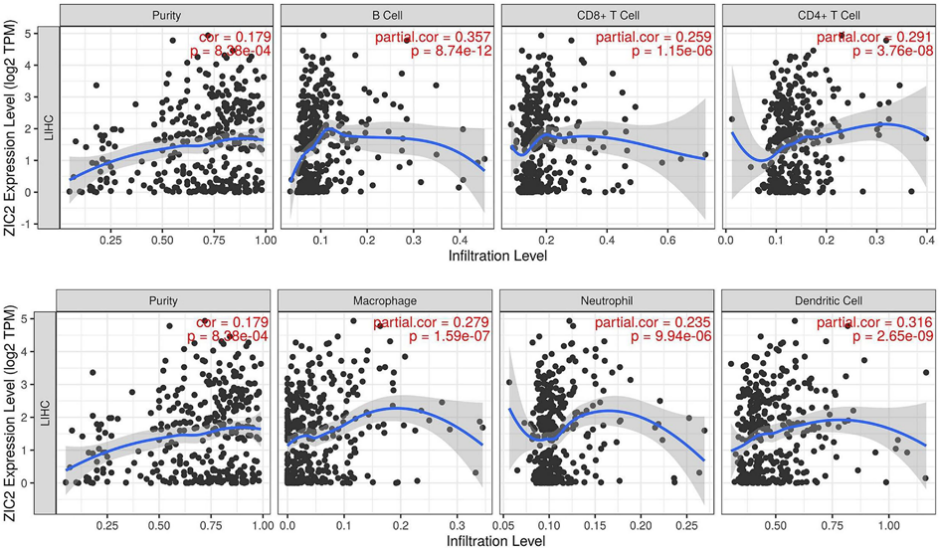
Relationship between *ZIC2* gene expression and immune infiltration in HCC (Cor > 0, *P*<0.001)

**Figure 7:**
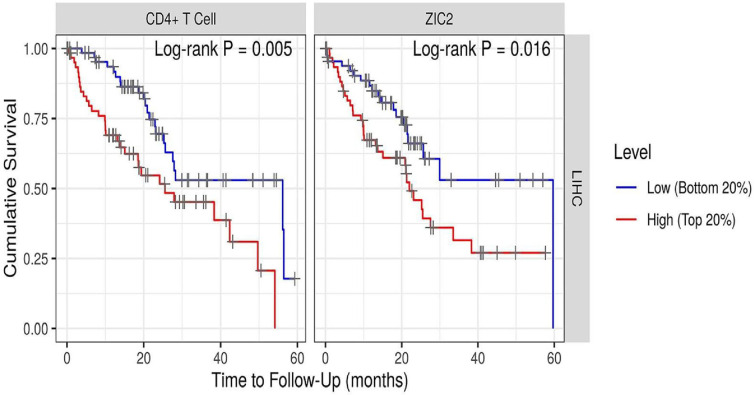
The effects of high expression of *ZIC2* mRNA in CD4+ T cells on the 5-year survival rate of patients with HCC

After *ZIC2* input in the TIMER database, a scatter diagram was generated and displayed, revealing the partial Spearman Rho values and statistical significance following purity corrections. The relative gene expression level of tumor purity is always presented on the leftmost panel (Figure 6), as genes highly expressed in the microenvironment are negatively correlated with tumor purity, whereas genes overexpressed in tumor cells are positively correlated. Surprisingly, the expression of *ZIC2* mRNA revealed a significant positive correlation with tumor purity, and with B cell, CD8+ T cell, CD4+ T cell, macrophage, neutrophil, and dendritic cell infiltration in the immune microenvironment of liver cancer (Timer database, Cor > 0, *P*<0.001, Figure 6). Moreover, via subgroup analysis, we found that the higher expression of *ZIC2* mRNA in CD4+ T cells was associated with a better 5-year survival prognosis compared with the lower expression, Nevertheless, this association needs to be further validated in the real world setting in large-scale clinical studies (Figure 7); however, a higher expression of ZIC2 mRNA was not associated with any significant differences in the 1-, 3-, and 5-year survival rates in B cells, CD8 + T cells, macrophages, neutrophils, or dendritic cells of patients with liver cancer (*P*>0.001).

## Discussion

Globally, primary liver cancer accounts for 6% of all cancers and 9% of all cancer deaths. It is the sixth most common cancer and has seriously endangered human life and health. In most patients, this disease is diagnosed in the middle and late stages because the early symptoms of this cancer are not clinically evident, which results in these patients not receiving the best surgical treatment opportunities. Therefore, the systematic treatment of advanced liver cancer has gained immense attention worldwide [24]. A landmark study revealed that the multitarget tyrosine kinase inhibitor sorafenib, the first approved drug for systemic treatment of patients with advanced HCC, resulted in a regrettable increase in the median OS of only 11 months [25]. Thus, breakthrough treatment of liver cancer is required. Moreover, our finding showed that immune cells are crucial in the progression of liver cancer, and their recruitment and function are profoundly affected by the tumor microenvironment via the interaction between tumor cells and the body’s immune response [26]. Furthermore, through these in depth studies we provided support for immunotherapy using immune checkpoint inhibitors as a promising novel therapy. This treatment approach has been widely studied in various tumor types including liver cancer because its mechanism mainly targets the regulatory pathways in T cells to release the body’s own immune response to attack the tumor. Recently, immune checkpoint inhibitors have been included in the liver cancer treatment arsenal [27]. In addition, through our in-depth study of tumor microenvironment and immunotherapy, we found that liver cancer is essentially an inflammation-related cancer. Sia et al. [28] performed inflammatory gene expression profiling, as well as pathological and immunohistochemical analysis of inflammatory cell infiltration using gene expression data from 956 liver cancer specimens, and identified specific signals associated with inflammatory activity. In 228 samples, the gene expression patterns were correlated with immune cell infiltration and the presence of immunomodulatory molecules, and thus proposed the concept of immune-specific liver cancer with specific biological characteristics. Its main features include the presence and activation of immune cells, enhanced cytolytic activity, protein expression of PD-1 and PD-L1, and the richness of genetic features that predict immunotherapy response [29]. Moreover, numerous studies have found that tumor-infiltrating lymphocytes, such as tumor-associated macrophages and tumor-infiltrating neutrophils, can affect the prognosis and efficacy of chemotherapy and immunotherapy [30]. The development of genomics technology has made an immense contribution to our understanding of carcinogenic mechanisms in the past decade, and has led to the identification of novel therapeutic targets. Incorporating genomic data into staging and treatment algorithms will have significant implications for predicting prognosis, guiding treatment protocols, and monitoring treatment response [31]. Thus, the identification of predictive biomarkers and novel targeted therapeutic methods through genomics technology combined with bioinformatics analysis will be crucial for the treatment of all tumors including liver cancer in the future [32]. In particular, the present study revealed that *ZIC2* gene expression was positively correlated with immune infiltrating cells in patients with liver cancer, and suggests that *ZIC2* can be used as a prognostic biomarker to evaluate low prognosis and high immune infiltration of liver cancer. Furthermore, this association may also indicate reactivity with immune checkpoint inhibitors and drugs associated with primary resistance. With further research, *ZIC2* may represent the gene target for predicting the response of liver cancer immunotherapy in the future.

In the present study, we found that *ZIC2* was associated with differential gene expression in liver cancer tissues and normal tissues using the GEPIA database. We also determined that *ZIC2* was highly expressed in breast, cervical, colorectal, and gastric cancers, using the Oncomine database. Moreover, differentially expression of ZIC2 was associated with the survival prognosis of patients with liver cancer as determined using the Kaplan-Meier Plotter database tool. The OS and PFS of patients with high *ZIC2* expression in liver cancer were significantly lower than those of patients with lower *ZIC2* expression.

In particular, this study revealed that *ZIC2* expression is associated with the levels of immune cell infiltration in liver cancer. More specifically, this expression was positively correlated with the immune infiltration of B cells, CD8+ T cells, CD4+ T cells, macrophages, neutrophils, and dendritic cells in patients with liver cancer. CD4+ T cell patients with high expression of *ZIC2* mRNA in liver cancer had a better 5-year survival prognosis, as analyzed through the Timer database.

In summary, the high expression of *ZIC2* gene indicates that the survival of patients with liver cancer is short and the prognosis is poor, and the correlation of these results suggest that *ZIC2* may exert a potential regulatory mechanism that is crucial to the recruitment and regulation of immune infiltrating cells in liver cancer. Moreover, *ZIC2* plays an important role in the progression, invasion, and metastasis of liver cancer. Therefore, we predict that *ZIC2* may be a crucial gene involved in the frontline response of immune cells, and may be associated with immune infiltration and prognostic biomarkers in patients with liver cancer. Nevertheless, to understand the prognosis and predictive biomarkers of liver cancer, additional basic research associated with data from the clinical setting are needed for appropriate clinical decision-making and to ultimately improve the prognosis of patients with liver cancer.

## Data Availability

Data associated with the present paper can be obtained by contacting the corresponding author.

## Abbreviations

DEG: differentially expressed gene
GEO: Gene Expression Omnibus
GEPIA: gene expression profiling interactive analysis
GO: gene ontology
HCC: hepatocellular carcinoma
LIHC: liver hepatocellular carcinoma
OS: overall survival
PFS: progress-free survival
TCGA: The Cancer Genome Atlas
TIMER: tumor immune evaluation resource
ZIC2: zinc finger protein 2 gene
